# Femtojoule optical nonlinearity for deep learning with incoherent illumination

**DOI:** 10.1126/sciadv.ads4224

**Published:** 2025-01-31

**Authors:** Qixin Feng, Can B. Uzundal, Ruihan Guo, Collin Sanborn, Ruishi Qi, Jingxu Xie, Jianing Zhang, Junqiao Wu, Feng Wang

**Affiliations:** ^1^ Department of Physics, University of California, Berkeley, Berkeley, CA 94720, USA.; ^2^ Materials Sciences Division, Lawrence Berkeley National Laboratory, Berkeley, CA 94720, USA.; ^3^ Department of Chemistry, University of California, Berkeley, Berkeley, CA 94720, USA.; ^4^ Department of Materials Science and Engineering, University of California, Berkeley, Berkeley, CA 94720, USA.; ^5^ Graduate Group in Applied Science and Technology, University of California, Berkeley, Berkeley, CA 94720, USA.; ^6^ School of Physical Science and Technology, ShanghaiTech University, Pudong District, Shanghai 201210, China.; ^7^ Kavli Energy NanoScience Institute, University of California, Berkeley, Berkeley, CA 94720, USA.

## Abstract

Optical neural networks (ONNs) are a promising computational alternative for deep learning due to their inherent massive parallelism for linear operations. However, the development of energy-efficient and highly parallel optical nonlinearities, a critical component in ONNs, remains an outstanding challenge. Here, we introduce a nonlinear optical microdevice array (NOMA) compatible with incoherent illumination by integrating the liquid crystal cell with silicon photodiodes at the single-pixel level. We fabricate NOMA with more than half a million pixels, each functioning as an optical analog of the rectified linear unit at ultralow switching energy down to 100 femtojoules per pixel. With NOMA, we demonstrate an optical multilayer neural network. Our work holds promise for large-scale and low-power deep ONNs, computer vision, and real-time optical image processing.

## Teaser

An energy-efficient and large-scale nonlinear optical device fills the gap to achieve computing in all-optical domain.

## INTRODUCTION

As all-purpose digital computation, particularly for artificial intelligence and deep neural networks, reaches an energy bottleneck, alternative physics–based computational architectures are attracting increasing attention (*1*–*13*). Among these, optical neural networks (ONNs) are a promising alternative due to their high parallelism, energy efficiency, and minimal latency (*14*–*17*). Highly parallel linear operations, such as matrix multiplications (*18*–*25*) and convolutions (*26*–*28*), can readily be implemented using nearly dissipationless linear optical transformations in ONNs. On this front, ONNs offer substantial energy savings per linear operation compared to cutting-edge all-digital counterparts (*24*, *29*–*32*). However, achieving an efficient optical nonlinearity poses inherent challenges (*33*, *34*), leading ONNs to often rely on hybrid systems that incorporate electronic nonlinear activations. These hybrid ONNs require preamplifiers and analog-to-digital converters to process weak optical signals that increase latency and power spent per operation (*15*). To realize deep ONNs with low energy consumption, the development of a sub-picojoule optical nonlinearity is crucial.

Recently, a “receiverless” approach has been proposed for energy-efficient optical modulation, which obviates the need for power-hungry electronics by in situ integration of a photodiode (PD) with an electro-optical modulator (EOM) (*32*, *35*). In this configuration, a portion of the input light generates photocarriers, which directly charge (or discharge) the EOM, thereby modulating the remaining part as the output. This process facilitates light self-modulation, with energy consumption that scales with the capacitance of the PD and the EOM. Following this approach, optical nonlinear operation with switching energy on the order of femtojoule per activation has been demonstrated in integrated photonics circuits by integrating femto-farad capacitance PDs and EOMs such as InGaAsP photonic crystals or micro-ring resonators. (*23*, *36*). However, integrated photonic devices face scalability challenges and lack compatibility with incoherent light, strongly restricting their use in large-scale ONNs in ambient light scenarios.

A free-space counterpart of the receiverless optical nonlinearity has the potential to address the scalability concerns by harnessing the immense parallel computing capabilities afforded by free-space light propagation. Further, free-space ONNs have compelling applications in object detection and sensing where conventional neural networks are routinely used to run inference on digitized camera images. In such applications, free-space ONNs could remove the need for the digitization step and run inference directly on the ambient light (*25*). Previously, liquid crystal (LC) light valves (LCLVs) have been developed for controlling a read beam with a write beam by placing a photosensitive film next to an LC EOM layer, with a dielectric mirror separating the two (*37*–*39*). With LCLVs, the sigmoid-like nonlinear dependence of the read beam intensity on the write beam intensity has been demonstrated and applied to the early research on ONNs (*40*, *41*). More recently, self-modulation of light has been realized by resistively coupling the LC layer to two-dimensional (2D) material phototransistor arrays, but the energy consumption is well above picojoule per operation (*42*). To the best of our knowledge, a femtojoule–rectified linear unit (ReLU) for self-activation of the input patterns—the predominant nonlinear function in contemporary deep neural networks—has never been realized.

In this study, we present an energy-efficient and highly parallel nonlinear optical microdevice array (NOMA) for free-space optical computation. Each pixel of the device contains a silicon (Si) PD capacitively coupled to an LC cell, allowing for nonlinear activations at the femtojoule scale. By leveraging the mature fabrication processes for Si-based integrated circuits and LC display technologies, our design readily enables the fabrication of devices with millions of pixels. Through the characterization of NOMA, we present an optical ReLU nonlinearity operating on an incoherent optical beam. Further, we demonstrate the practical applications of this optical ReLU in two optical processing tasks: real-time image contrast enhancement and nonlinear activation within a multilayer ONN (ML-ONN).

## RESULTS

### Device structure and working principle

**Fig. 1.**
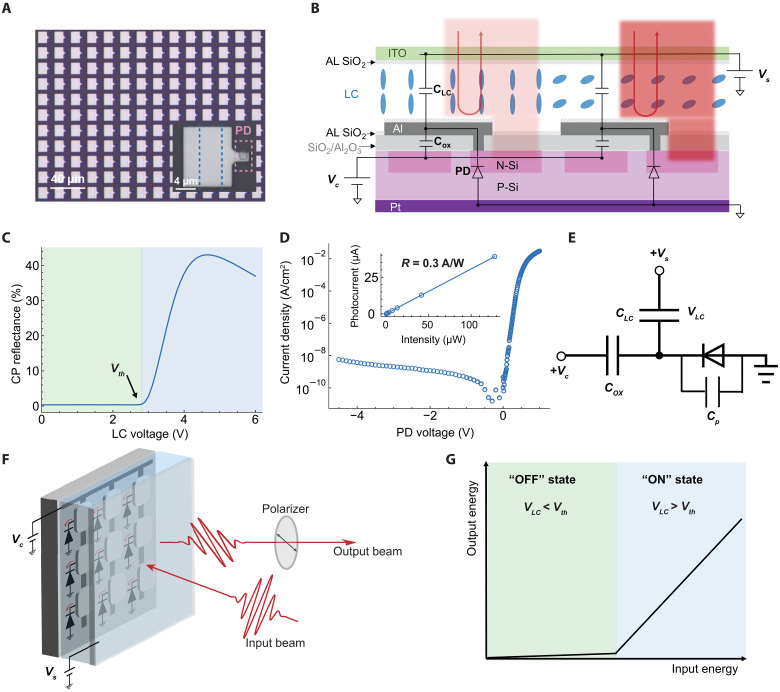
Device structure and working principle. (**A**) Microscope image of NOMA. The inset shows a single pixel, depicting the metallic region that is the Al mirror. The n-doped region outlining the PD is indicated by pink dashed lines, and the additional n-doped region underneath the Al mirror is outlined by the blue dashed lines. (**B**) Cross section of NOMA pixels. The device consists of an LC layer, represented by blue rods between an ITO-covered glass and a Si substrate with PDs. Both substrates are coated with a SiO_2_ for LC alignment (AL). Upon illumination, PD charges the LC cell, leading to rotation of the LC molecules and modulation of reflected light through LC birefringence. (**C**) Characterization of the LC birefringence using voltage-dependent CP reflectance of the LC cell with λ = 680 nm, showing a threshold voltage (*V*_*th*_) at 2.8 V. (**D**) Current-voltage relation of the Si PD under dark conditions. The measured PD is the one formed between the auxiliary n-doped region and substrate. The insert shows the relation between photocurrent and light intensity, indicating a responsivity (*R*) of 0.3 A/W. (**E**) Equivalent circuit for a NOMA pixel. *C*_*LC*_ denotes the capacitance between the Al mirror and the ITO electrode. *C*_*ox*_ denotes the capacitance between the Al mirror and the n-doped region beneath it. *C*_*p*_ represents the junction capacitance. (**F**) Configuration of NOMA for light self-modulation. The input beam is a linearly polarized pulse, and the output beam is the CP reflected pulse. (**G**) ReLU-like input-output relationship. For incident light with small pulse energy, the NOMA remains in the OFF state and the transmitted light energy through the polarizer (output energy) is suppressed. For incident light with large pulse energy, the NOMA is switched to the ON state, characterized by a high CP reflectance.

We fabricated an NOMA comprising 750 × 700 pixels, each with a dimension of 20 μm by 20 μm (Fig. 1A). The Materials and Methods of the supplementary text describe the detailed structure and the fabrication process for the entire device. Figure 1B schematically illustrates the structure of the NOMA. Within each pixel, there is a Si PD connected to an Al mirror while the LC fills the gap formed between the Si substrate and the indium tin oxide (ITO)–coated glass. The Si substrate is grounded, while a source voltage *V*_*s*_ is applied to the ITO electrode. An additional n-doped region beneath the Al mirror serves as a global electrode for a control voltage *V*_*c*_. Both *V*_*s*_ and *V*_*c*_ are nonnegative to ensure a reverse-biased PD.

For optimal optical modulation, we use a vertically aligned nematic LC with a large contrast ratio (*43*, *44*). We characterize the optical intensity modulation of the LC cell using cross-polarized (CP) reflectance as a function of *V*_*LC*_ (Fig. 1C). In this configuration, the LC cell acts as a tunable half-wave plate placed between two crossed polarizers. At low bias, the LC cell appears dark as LC molecules are aligned parallel to the propagation direction of the incident beam and no change in the polarization state of the incident beam occurs. At a threshold bias (*V*_*th*_) of 2.8 V, the LC molecules start to tilt, causing the incident beam to attain ellipticity and the LC cell appears brighter. The measured value closely approximates the theoretical value of 2.2 V predicted by the Freedericksz transition theory (*45*, *46*), considering the mismatch in electrode work functions (0.4 V, between Al and ITO). As the bias is increased above *V*_*th*_, a clear maximum in reflectance occurs where the polarization of the incident beam is completely rotated to the perpendicular polarization. The LC cell exhibits a contrast ratio of 120, providing a broad optical modulation range.

As the Si PD provides in situ optical-to-electrical feedback, low dark current and high optical responsivity are crucial for energy-efficient nonlinear operations. By using an Al_2_O_3_ passivation layer (*47*), we achieve a low-junction dark current of 10 nA/cm^2^ (Fig. 1D). Given that the PD area in each pixel is approximately 100 μm^2^, the dark current per pixel is around 10 fA. We measure the responsivity of our PD as 0.3 A/W (λ = 680 nm), indicating efficient collection of photocarriers.

To elucidate the dynamics of the optical ReLU nonlinearity, we introduce a simple circuit model depicted in Fig. 1E, which consists of three main elements: (i) an LC capacitor (*C*_*LC*_), which forms between the Al mirror and the ITO electrode; (ii) a Si PD, which can be described by an ideal diode in parallel with a junction capacitor *C*_*p*_; (iii) an oxide dielectric capacitor (*C*_*ox*_), which forms between the Al mirror and the additional n-doped region. We list the estimated capacitance values of each in table S1. Upon illumination, part of the incident light is reflected by the Al mirror, while the rest is absorbed by the Si PD. The photocarriers generated in the Si PD accumulate on the Al mirror, altering the voltage across the LC cell (*V*_*LC*_). Consequently, the orientation of the LC molecules changes, which then modulates the reflected light through the LC’s birefringence (Fig. 1F). Under dark or weak illumination, the *V*_*LC*_ remains below *V*_*th*_, so the NOMA remains in the “OFF” state with a low CP reflectance. Thus, the energy of the CP-reflected light (output energy) is suppressed. On the other hand, for incident light with a high pulse energy, the LC capacitor is charged above *V*_*th*_, so the NOMA is switched to the “ON” state, characterized by a high CP reflectance. In this state, the output energy exhibits a linear dependence on the input energy. This characteristic behavior mimics the ReLU function, where the output remains zero for low input values and increases linearly with higher inputs (Fig. 1G).

### Optical switching dynamics

**Fig. 2.**
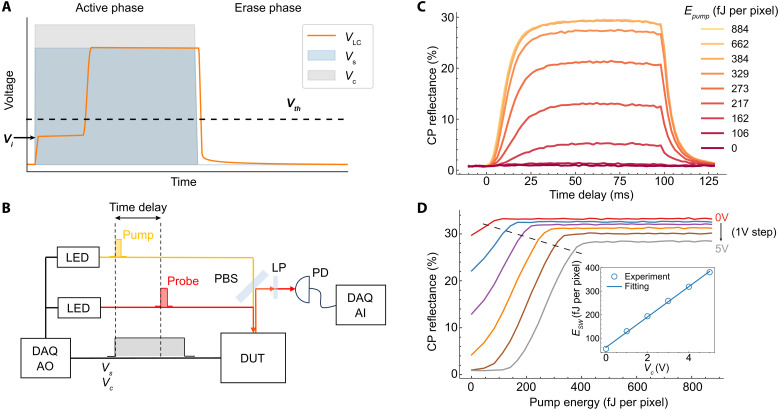
Optical switching dynamics. (**A**) Waveform of *V*_*s*_, *V*_*c*_, and the resulting LC capacitor voltage *V*_*LC*_. Both *V*_*s*_ and *V*_*c*_ are synchronized square waves, characterized by an active and erase phase. In the active phase, an intense optical pulse can charge the *V*_*LC*_ from the below-threshold initial value *V*_*i*_ to above-threshold value *V*_*s*_, resulting in NOMA switching from the OFF to the ON state. In the erase phase, the LC capacitor is discharged, setting it to the default OFF state. (**B**) Illustration of the pump-probe experiment (see fig. S3A for an optical layout), where pump (λ = 630 nm) and probe pulses (λ = 680 *nm*) from LEDs are directed onto NOMA. Using analog outputs (AO) of a data acquisition card (DAQ), the light pulses are synchronized with the applied bias such that the pump arrives at the rising edge of the bias, while the probe pulse is time-delayed. Dynamics in the CP reflectance of NOMA is measured using a polarized beam splitter (PBS) with a long-pass filter (LP) in a wide-field microscope geometry. The total intensity of the reflected light is measured using a PD and digitized using DAQ analog inputs (AI). (**C**) The dynamics of CP reflectance of NOMA at increasing pump energies (lighter shades) *E*_*pump*_ (*V*_*s*_ = 4 V and *V*_*c*_ = 4.5 V) largely follow the outline of the voltage pulse with a rise and fall time limited by the LC. (**D**) *E*_*pump*_-dependent CP reflectance at different *V*_*c*_ (*V*_*s*_ fixed at 4 V). The insert illustrates the expected linear relationship between switching energy *E*_*sw*_ and *V*_*c*_, showing a minimum *E*_*sw*_ at 60 fJ/pixel.

We periodically operate NOMA between an active and erase phase, as illustrated in Fig. 2A. During the active phase, the LC capacitor first charges to an initial voltage *V*_*i*_, which is determined by the capacitance divider: $V_{i}=\frac{C_{p}+C_{\mathrm{ox}}}{C_{\mathrm{tot}}}V_{s}-\frac{C_{\mathrm{ox}}}{C_{\mathrm{tot}}}V_{c}$, where $C_{\mathrm{tot}}=C_{\mathrm{LC}}+C_{\mathrm{ox}}+C_{p}$. This charging process happens on a timescale of $\tau_{1}=R_{s}C_{\mathrm{tot}}$, where $R_{s}$ is the series resistance of bulk silicon and the contact. Given that $R_{s}$ is approximately ∼10^6^ ohm and $C_{\mathrm{tot}}$ is around 20 fF, τ_1_ occurs on the timescale of microseconds. Under dark condition, the LC capacitor slowly charges to *V*_s_ where the charging time (τ_2_) is set by the dark current *I*_*R*_ of the Si PD. Given that *I*_*R*_ is in the order of 10 fA/pixel, τ_2_ is in the order of seconds. In our experiments, we apply a square wave of *V*_*s*_ and *V*_*c*_ with a period *T* of milliseconds, which is determined by the LC’s response time. Considering that τ_1_ ≪ *T*/2 ≪ τ_2_, the LC voltage remains relatively constant at *V*_*i*_ under dark condition during the *T*/2 ms active phase. Under light illumination, the photocarriers generated in the PD lead to a rapid charging of the LC capacitor to *V*_s_. We maintain *V*_*s*_ above *V*_*th*_ and adjust *V*_*c*_ so that *V*_*i*_ remains below *V*_*th*_, ensuring that the LC is in the OFF state without light but can transition to the ON state under sufficient light illumination. In the erase phase, we keep *V*_*s*_ below *V*_*th*_ and set *V*_*c*_ to 0 V, dissipating the accumulated charges in the LC capacitor and reverting the device to the default OFF state.

The optical switching energy *E*_*sw*_ is determined by the amount of photocarriers (*Q*_*ph*_) needed to fully charge the LC and oxide capacitors, which can be estimated from our circuit model as

$$E_{\mathrm{sw}}=\frac{Q_{\mathrm{ph}}}{\alpha }=\frac{C_{\mathrm{LC}}\Delta V_{s}+C_{\mathrm{ox}}\Delta V_{c}}{\alpha }$$ (1)

where α is the optical-to-electrical coefficient and Δ*V*_*s*_ and Δ*V*_*c*_, respectively, denote the changes of *V*_*s*_ and *V*_*c*_ between the active and erase phases. Considering the responsivity of silicon PD (0.3 A/W), fill factor of Al electrode, and transmission loss through the ITO layer, α is approximately 0.1 C/J. Given that both *C*_*ox*_ and *C*_*LC*_ are in the range of femtofarad, *Q*_*ph*_ is estimated to be in the tens of femtocoulombs. Thus, the optical switching energy for each pixel is calculated to be in the hundreds of femtojoule range. Regarding the electronic energy consumed during the switching process, it is essentially the work done by the voltage sources, which can be calculated as $E_{\text{electronic}}=C_{\mathrm{LC}}\Delta V_{s}^{2}+C_{\mathrm{ox}}\Delta V_{c}^{2}$. It indicates that the electric switching energy is on the same scale of the optical switching energy.

To investigate the dynamics of the optical switching process, we carried out pump-probe experiments capable of optically probing the device dynamics at the characteristic time scale of the LC molecules (Fig. 2B). We use short pulses (2-ms duration) from colored light-emitting diodes (LEDs) for both pump and probe lights. The pump light is synchronized with the electrical signal’s rising edge, which charges the LC cell and initiates the optical switching. The probe light measures the CP reflectance of the device as a function of time delay between the pump and probe. To ensure that the observed dynamics are only from the pump-induced changes, we use a probe pulse energy (80 fJ per pixel) below the threshold energy of NOMA and use probe pulse durations (2 ms) much shorter than the LC response time (~10 ms). We map the CP reflectance as a function of *V*_*s*_, *V*_*c*_, and pump energy (*E*_*pump*_). Using these results, we quantify the capacitances in our circuit model and identify the optimal conditions for efficient optical nonlinearity.

Figure 2C shows time traces of CP reflectance at increasing pump energies at *V*_*s*_ = 4 V and *V*_*c*_ = 4.5 V. For subthreshold pump energies (i.e., 0 and 106 fJ per pixel), CP reflectance traces show a negligible increase after pumping, indicating that the device remains in the OFF state. In contrast, at a higher pump energy, the CP reflectance increases notably after pumping and reaches a plateau. With increasing pump energy, the plateau value rises from 0.8% to a maximum of 29%, showing a large modulation range of 35 between the OFF and ON states.

We further characterize the optical switching by measuring the CP reflectance at a fixed probe time delay at 60 ms, at which point the CP reflectance reaches its plateau. Figure 2D displays the measured CP reflectance as a function of *E*_*pump*_ at different *V*_*c*_. The derived switching energy *E*_*sw*_ (defined as the pump energy at which the CP reflectance reaches 95% of its saturation level) shows a linear dependence on *V*_*c*_, consistent with our circuit model (Eq. 1). From the slope and intersection of the linear fit, we derive *C*_*ox*_ = 6.5 fF per pixel and *C*_*LC*_ = 5.8 fF per pixel, which are close to their estimated values (table S1).

### Femtojoule optical ReLU for image contrast enhancement

**Fig. 3.**
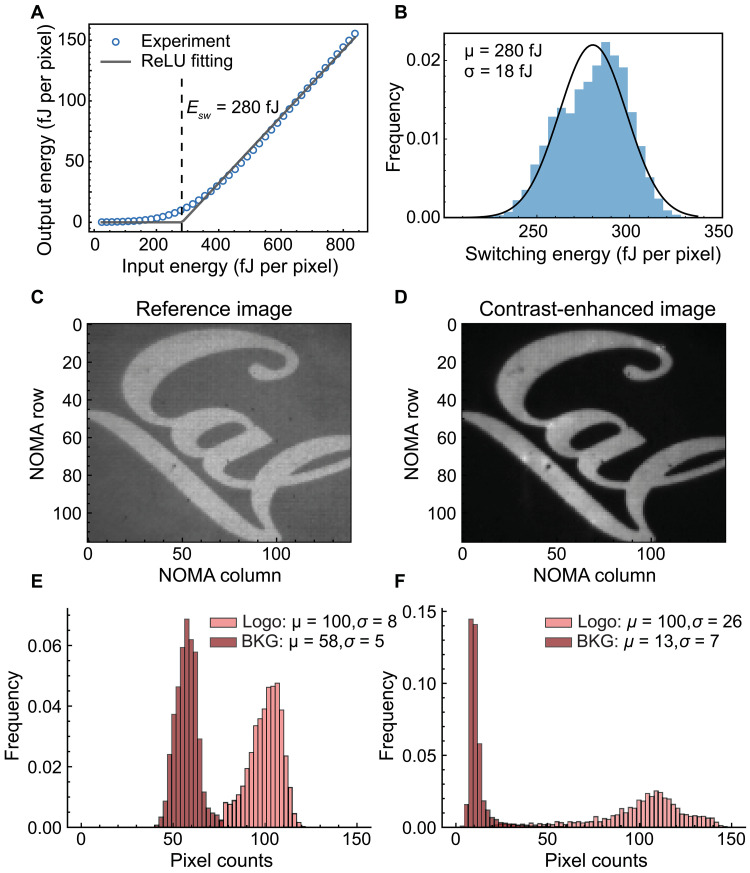
Demonstration of the optical ReLU and image contrast enhancement. (**A**) Average output energy versus input energy across more than 10,000 NOMA pixels, showing a nonlinear dependence, which is fitted with an ReLU function (gray line) with a switching energy *E*_*sw*_ of 280 fJ. The histogram of *E*_*sw*_ (**B**) shows a normal distribution with a mean (μ) of 280 fJ and an SD (σ) of 18 fJ. The reference (**C**) and contrast-enhanced (**D**) image reflected from the NOMA, which is operated under a linear response and the ReLU response, respectively. The input image is a “*Cal*” logo, which consists of two regions: the logo region, chosen to be bright, and the background, chosen to be dark. Investing the two regions separately in terms of their pixel values reveals histograms with a mean (μ) and SD (σ) value for the reference image (**E**) and contrast-enhanced image (**F**), demonstrating four-time improvement in the image contrast.

We demonstrate the optical ReLU function using a single but longer LED light pulse (50 ms) synchronized with the rising edge of the electrical pulses. The voltages are set at *V*_*s*_ = 4 V and *V*_*c*_ = 5.5 V. The distinct signature of the optical nonlinearity is depicted in Fig. 3A, where the relationship between CP reflected light energy (output energy) and incident light energy (input energy) resembles an ReLU function with a switching energy of 280 fJ per pixel. At low input energies, the reflectivity of NOMA is marginal at around ~1%. At higher input energies, the reflectivity increases to 24% and saturates. The saturated reflectivity is limited by the fill factor of the Al electrode and the optical losses at the interfaces of the device stack. Analogous to the response to short pulses, we can manipulate the switching energy value of the ReLU function by adjusting *V*_*c*_ (fig. S4). This additional tunability is useful in applications where the optical input varies dynamically, enabling the device to maintain optimal performance across various scenarios.

To further investigate the response at the individual pixel level, we capture wide-field images of NOMA and track the pixel-by-pixel dynamics. We segment the wide-field images into a regular grid, each grid containing only one pixel of NOMA (fig. S5A). Figure 3B shows the statistics from 10,201 NOMA pixels. The ReLU function has a normal distribution of the switching energy with a mean value of 280 fJ and an SD of 18 fJ. In applications such as ONNs, some degree of heterogeneity in the response of individual pixels is acceptable and even leveraged through hardware-aware fine-tuning of the models (*48*, *49*). Yet, achieving uniform and consistent response across many pixels is generally desirable as cascading errors can potentially impede computation, especially for deep neural networks.

To demonstrate the ReLU functionality and large-scale uniformity, we performed a contrast enhancement task of a binary grayscale image through interaction with more than 15,000 NOMA pixels. As our baseline, we capture an image reflected from NOMA under the linear response (Fig. 3C). We ensure the linear response of the device by setting *V*_*s*_ = −4V to forward bias the Si PD, which maintains the LC cell in the ON state. To capture the contrast-enhanced image (Fig. 3D), we set *V*_*s*_ = 4 V and *V*_*c*_ = 5.5 V to ensure that NOMA is under an ReLU response. Compared with the dark regions of the reference image, the contrast enhanced image shows a darker background while the bright regions retain their average brightness. Quantitatively, the image processed with nonlinearity exhibits a contrast four times greater than that of the reference image (Fig. 3, E and F). The ability of NOMA to selectively amplify the contrast of specific image regions showcases its potential in applications such as real-time image processing and optical edge computing. In these cases, the nonlinear layer prunes or maintains connections between successive layers in the network. In spirit, this contrast enhancement task can be thought of as one such application where we already demonstrate more than 10,000 nonlinear connections with potential to expand into deep neural networks with more than one hidden layer.

### ML-ONN with ReLU activations

**Fig. 4.**
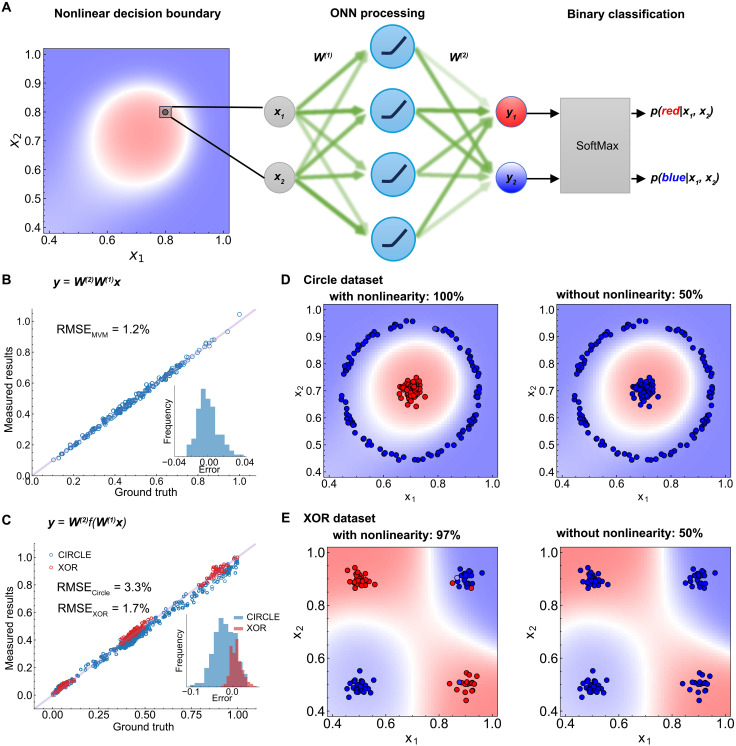
ML-ONN for binary classification. (**A**) Schematic illustration of the ML-ONN used for the binary classification task. The input to the ML-ONN consists of coordinates (*x*_1_, *x*_2_) of a point in 2D space, separated into two classes by a nonlinear boundary. The implemented ML-ONN is a two-layer fully connected neural network with optical nonlinear activation using the NOMA with ReLU response shown in Fig. 3A. (**B**) Performance of the linear operation of the ML-ONN shown as a scatter plot of measured outputs against ground truth of 80 random MVM. In an ideal implementation, the measured output of the MVM and the ground truth fall on a line with slope of 1, marked by the purple line in the figure. The inset panel is an error histogram illustrating the scatter around this ideality line. The histogram is characterized by a root mean squared error (RMSE) of 1.2%. (**C**) Scatter plots characterizing the RMSE of the full ML-ONN, including the nonlinear activation between MVMs, along with the error histogram (inset). RMSE rates for two classification tasks on two datasets (Circle and XOR) are characterized as 3.3% and 1.7% respectively. The distribution and the underlying decision boundaries of the Circle dataset and the XOR dataset are shown in (**D**) and (**E**), respectively. In both cases, the classification accuracy is >97% when the NOMA is operated under an ReLU response, while the accuracy is 50% (random chance) when it is operated under linear response conditions.

We highlight the role of the ReLU nonlinearity by demonstrating an ML-ONN. The implemented ML-ONN consists of two fully connected linear layers linked by the NOMA, serving as the nonlinear activation layer. We leverage the ML-ONN to tackle two distinct binary classification tasks characterized by nonlinear decision boundaries. Figure 4A illustrates the ground truth for one such boundary, separating the 2D space defined by the input vector ***x*** = (*x*_1_, *x*_2_) into red and blue regions by a circular decision boundary. Our network is configured with two inputs, four hidden neurons, and two output neurons with the goal of learning these nonlinear decision boundaries. The ML-ONN maps the input vector ***x*** to an output vector ***y*** = (*y*_1_, *y*_2_) through two transformation matrices and one ReLU nonlinear activation. We determine the class of the input point (red or blue) by comparing the magnitudes of *y*_1_ and *y*_2_ or, more precisely, calculating the posterior probabilities with the SoftMax function.

In our optical implementation, we encode ***x*** into light intensity and the weight matrices [*W*^(1)^ and *W*^(2)^] into the reflectivity of spatial light modulators (SLMs). We use an optical fan out to implement matrix vector multiplications (MVMs) (*25*). The Supplementary Materials contain a detailed description of the optical layout (fig. S7) as well as the test and validation of the implemented optical hardware in supplementary section S3.

We first quantify the operation precision of the linear and nonlinear layers in the ML-ONN. We evaluate the linear operation capabilities of our ML-ONN by performing random MVMs on the SLMs. Figure 4B shows the measured light intensities after the multiplication operation versus the theoretical values. On the basis of this comparison, we determine a root mean square uncertainty of 1.2% in our optical MVM implementation. This error rate indicates an effective calculation precision of 6.2 bits for our linear operations.

We next performed an optical inference experiment with the complete ML-ONN depicted in Fig. 4A across two binary classification datasets, namely, Circle and XOR. Figure 4C shows the measured light intensity versus the theoretical values. The comparison shows that the root mean square uncertainty increases to 1.7 ~ 3.3% when we include a nonlinear activation through NOMA, resulting in an effective calculation precision of 5 bits. Figure 4 (D and E) illustrates the inference results of the Circle and XOR classifications, respectively, where the shaded regions show the underlying decision boundaries. For XOR and Circle datasets, the test accuracy stands at 97 and 100%, respectively. In contrast, the inference accuracy without nonlinearity is only 50% for both datasets. The poor accuracy in the absence of nonlinearity is expected as the network without optical nonlinearity essentially functions as a linear regression model, incapable of capturing the inherent nonlinear decision boundary of these datasets.

## DISCUSSIONS

In this study, we implemented an NOMA for an energy-efficient optical nonlinearity by integrating Si PD and LC EOM at a single-pixel level. The NOMA achieves an optical ReLU function with switching energy down to 100 fJ across more than half a million pixels. We further demonstrated NOMA’s energy efficiency, uniform nonlinear response, and compatibility with incoherent light through an image contrast enhancement task and highlighted the optical ReLU function in a binary classification task for deep ML-ONNs. In contrast with the state-of-the-art analog optoelectronic neural networks (*17*), NOMA eliminates the need for shuttling signals back and forth between optical and electrical domains, which should enable neural networks with more than one hidden layer in an energy-efficient manner. Further improvements to the switching energy can be achieved by decreasing EOM and PD capacitance, which is ultimately limited by the circuit Johnson noise. For instance, by reducing the pixel pitch from the current 20 to 3 μm (comparable to the state-of-art LC on silicon technology) and using a smaller-capacitance P-I-N junction as the PD, the capacitance of a single pixel can be as low as 100 aF, enabling optical modulation at sub-femtojoule switching energies.

We report switching times on the order of milliseconds for our energy-efficient nonlinearity. At these switching times, an NOMA-based ONN can be used as a drop in energy-efficient replacement for digital neural networks in applications where the inference task is frame rate limited. Such situations arise in a broad range of image recognition tasks, including applications in autonomous vehicles and facial recognition. Furthermore, an NOMA-based optical nonlinearity could be used in image compression as an efficient optical encoder layer that alleviates bandwidth challenges associated with large images. More excitingly, an energy-efficient optical nonlinearity, such as NOMA, could enable the development of optical neuromorphic computation platforms that mimic biological functions, such as visual perception.

The NOMA initially addressed a fundamental challenge of nonlinearity within the all-optical neuromorphic computing framework, which generally requires high energy efficiency, scalability, and broadband compatibility. We believe that our approach could catalyze the development of large-scale deep ONNs for intelligent edge computing and sensing in the future.

## MATERIALS AND METHODS

Figure S1A is the photograph image of a completed NOMA with 750 × 700 pixels. Figure S1B illustrates the design of a 20 μm–by–20 μm NOMA pixel. Each pixel contains a rectangular Al mirror that occupies approximately *A*_*Al*_ = 240 μm^2^ or 60% of the total pixel area. The Al mirror is connected to a Si PD through a 4-μm^2^ contact via. The area of the Si PD is *A*_*PD*_ = 100 μm^2^. Beneath the Al mirror, a 6-μm-wide additional n-doped region extends across the column of pixels and connects to a common Al electrode at the edge of the device. The device’s vertical structure, displayed in Fig. 1B, consists of an ITO glass and a Si backplane, forming an LC cell with an approximate thickness of *d* = 3 μm. Given the LC’s refractive index anisotropy (Δ*n* = *n*_*e*_ − *n*_*o*_) of about 0.1, the retroreflected light’s maximum path difference (Δ*L*) between the ordinary (o) and extraordinary (e) light is roughly 0.6 μm (Δ*L* = 2*d*Δ*n*), corresponding to a 1.8π phase retardation for a wavelength of 670 nm. This phase shift is sufficient for a full-range intensity modulation, which typically requires a phase modulation between 0 and π.

We used conventional planar fabrication techniques for the silicon substrate. To define the Si PD, we doped a 6-inch (15.24-cm) p-type Si wafer (ρ = 10 to 20 ohm·cm, Silicon Valley Microelectronics, USA) using phosphorus thermal diffusion under a POCl_3_ atmosphere at 840°C. We next established a dielectric stack on the Si substrate through the deposition of 15 nm Al_2_O_3_ at 250°C by atomic layer deposition and 530 nm SiO_2_ at 350°C by plasma-enhanced chemical vapor deposition. Notably, the Al_2_O_3_/Si interface hosts a substantial built-in charge density, which effectively passivates minor carrier recombination at the surface, reducing the surface leakage current of the Si PD (*50*). Following the formation of the dielectric stack, the wafer underwent a patterning and etching process to create contact vias for Al mirrors. This was followed by 200-nm Al sputtering and patterning. Then, we established an ohmic contact to the p-type Si using 100-nm Pt that was sputtered on the back side of the wafer (*51*). We used the ITO glass (MSE Supplies LLC) with a sheet resistance of around 30 to 60 ohm/sq. We coated both the silicon and ITO substrates with 40-nm SiO_2_ via oblique e-beam evaporation, which served as the alignment layer for the LC (*52*). We bonded the Si and ITO pieces together using an ultraviolet (UV) curing adhesive (OG142, Fiber Optic Center, USA). We controlled the cell gap using microspheres (Micropearl SP-203, Sekisui Chemical Co. Ltd., Japan) with a diameter of 3 μm, which were placed along the periphery of the chip within the UV adhesive. We filled the sealed cell through a small fill port at the edge of the bonded chips with nematic LC (LC-VAST14, INSTEC, USA) using the capillary effect to uniformly form the LC layer. Last, we sealed the fill port using the same UV adhesive, completing the assembly process.
