# Supplementary material for: Femtojoule optical nonlinearity for deep learning with incoherent illumination
Source: Sci Adv. 2025 Jan 31;11(5):eads4224. doi: 10.1126/sciadv.ads4224 (PMC11784804; doi:10.1126/sciadv.ads4224)
Supplement: Supplementary file 1 — Supplementary Text S1 to S3 Figs. S1 to S9 Tables S1 and S2 [file sciadv.ads4224_sm.pdf]

Science Advances
AAAS

Supplementary Materials for

Femtojoule optical nonlinearity for deep learning with incoherent illumination

Qixin Feng *et al.*

Corresponding author: Feng Wang, fengwang76@berkeley.edu

Sci. Adv. **11**, eads4224 (2025)
DOI: 10.1126/sciadv.ads4224

This PDF file includes:

Supplementary Text S1 to S3
Figs. S1 to S9
Tables S1 and S2

Supplementary Text

S1-Working Principle of NOMA.

(a) Circuit Model: As shown in Fig. S2, we proposed a lumped-element circuit model to explain the working principle of NOMA. In this model, the liquid crystal (LC) capacitor is connected to the cathode of the silicon photodiode (Si PD). A positive source voltage V_s is applied to the LC capacitor while the anode of Si PD is grounded, ensuring the Si PD is reversed biased. A control voltage V_c is used to control the initial voltage of the LC capacitor through the capacitive coupling of an oxide capacitor.

We periodically operate NOMA in the active phase and the erase phase. In the active phase, we set $V_s > V_{th}$ and $V_c > 0$. Without illumination, the LC capacitor initially charges to $V_i = \frac{C_p+C_{ox}}{C_{tot}} V_s - \frac{C_{ox}}{C_{tot}} V_c$, which is determined by the capacitance divider. To ensure that the voltage across the LC remains below the threshold V_{th}, we maintain $V_c > \frac{C_p+C_{ox}}{C_{ox}} V_s - \frac{C_{tot}}{C_{ox}} V_{th}$, preventing the LC molecule rotation. As a result, the NOMA remains in "OFF" state with a low cross-polarized (CP) reflectance. As shown in Fig. S2A, the pre-charge current flows from the voltage sources into the capacitors. With illumination (Fig. S2B), the photocurrent generated by the Si PD drains the junction capacitor, transferring charge to the LC capacitor. As the LC capacitor voltage surpasses V_{th}, LC molecules begin to rotate, thus the NOMA is switched to "ON" state with high CP reflectance. In the erase phase, we reduce V_s below V_{th} and set V_c to 0V. A forward biased pn junction current discharges the voltage of LC capacitor and oxide capacitor to V_s and ground, respectively. During this process, the discharge current flows from the capacitors back to the voltage sources. As a result, NOMA is reset to "OFF" state, as illustrated in Fig. S2C.

(b) Capacitance Estimation: Based on device geometry, we estimated the capacitance for each component of the pixel: C_{LC}, C_{ox} and C_p. For the LC capacitor formed between the Al mirror and the ITO electrode, the area A_{LC} is approximately $240\mu\text{m}^2$ with a thickness d_{LC} of $3\mu m$. The dielectric constant ε_{LC} varies between 3.7 and 8.6 as the orientation of LC molecule changes from parallel to perpendicular to the electric field, resulting C_{LC} value ranging from 2.6 fF to 6.1 fF. The influence of the alignment layer is considered negligible because its thickness is only 3% of the LC gap.

For the oxide capacitor, which forms between the Al mirror and the underlying additional n-doped region, the area A_{ox} is about $100\mu\text{m}^2$. The total dielectric stack thickness is characterized by an effective SiO_2 thickness: $d_{eff} = d_{\text{SiO}_2} + \frac{\varepsilon_{\text{SiO}_2}}{\varepsilon_{\text{Al}_2\text{O}_3}} d_{\text{Al}_2\text{O}_3} = 0.54\mu\text{m}$. Then the estimated C_{ox} is 6.4 fF.

Using the abrupt junction model, we can estimate the capacitance of Si PD. Given the substrate resistivity of $10 - 20\Omega\text{cm}$, the p-type doping concentration is around $N_a = 10^{15}\text{cm}^3$. The peak doping concentration of phosphorus thermal diffusion is around $N_d = 10^{20}\text{cm}^3$. Thus, the junction build-in potential $V_{bi} = 0.9\text{V}$. At zero bias, the junction depth x_j is derived to be $1.1\mu\text{m}$. The junction area comprises the top-view doping area of $100\mu\text{m}^2$, and a periphery area calculated as the product of the doping area's perimeter and junction depth. Thus, the total junction area is approximately $140\mu\text{m}^2$, and the estimated junction capacitance at zero bias is 12 fF. We summarize these capacitances in table S1.

S2-Optical Nonlinearity Experiments.

(a) Optical Layout: We ran a suite of experiments on NOMA aimed at demonstrating its nonlinear properties using the optical setup illustrated in Fig. S3(A). The optical setup contains two beam paths (named pump and probe) that use colored Cree LEDs (λ_{pump}= 630nm and λ_{probe}= 680nm) as their light source. Each LED is driven by a laser diode driver (Thorlabs, LDC205C) which can be modulated with an analog input. We used a beam splitter to combine the pump and probe pulses. Using the transmitted beam path through this beam splitter, we monitored the incident probe intensity on NOMA using a photodiode (Thorlabs, SM05PD1A). The pump pulse was focused onto the back focal plane of a 4X microscope objective (AmScope Plan 4/0.10) to create a uniform illumination across the field of view. The probe beam path contained a reflective spatial light modulator (SLM, SDE1024, Cambridge Correlators) in an intensity modulation configuration. A uniform bright image was displayed on the SLM and image onto NOMA using a lens and the same 4X microscope objective. The light reflected by NOMA is collimated by the 4x objective and reflective by a polarized beam splitter (PBS). The CP reflected light can be either imaged onto a CMOS camera (Allied Vision, 1800) or focused onto a photodiode (Thorlabs, SM05PD1A). The pump beam is blocked by a long-pass filter.

(b) Pump-probe Measurements: We periodically drive the NOMA in the active and the erase phase by applying a square wave of V_s and V_c. The pump light is synchronized with the rising edge of the voltages and the probe light is time-delayed relative to the pump light. The CP reflected light intensity can be measured by either the CMOS camera or a photodiode. We controlled the time delay between the arrival time of the pump and probe pulses as well as their duration (2ms) and intensity using a data acquisition card (National Instruments, NI-9264). We used the same data acquisition card to generate the voltages necessary for NOMA operation (V_c and V_s) as well as a camera trigger pulse synchronized to the probe arrival time. We set the exposure time of the camera to match the probe pulse duration. For a given time delay, we operated NOMA at 5Hz for 0.6 second which constitutes 3 "ON"- "OFF" cycles. The results of the pump-probe experiments for time delays that span the entire "ON" cycle are shown in Fig. 2(C) of the main text. A longer time trace that contains three periods of active and erase phase is shown in Fig. S3(B).

(c) Tunable Optical ReLU with Single Pulse: For single pulse nonlinearity experiments that show the all-optical ReLU response (such as Fig. 3(A) of the main text) we used a similar optical setup to the one outlined for the pump-probe experiments, but we only focused on the probe beam path. In this case, we used 50ms as the probe pulse duration and monitored the CP reflected light energy vs incident energy using the photodiodes. As shown in Fig. S4(A), the reflected vs incident energy (input-output relationship) shows a clear dependence on the control voltage (V_c) at a source voltage (V_s) of 4V. We observe that at low V_c, the input-output relationship exhibits near-linear behavior. An increase in V_c leads to a notable suppression of output energy, particularly at low input energy levels. To quantitatively describe this optical nonlinearity, we employed an Exponential Linear Unit (ELU) function to fit the experimental data. The ELU function is expressed as:

$$ELU(x) = \begin{cases} R(x - E_{sw} + E_0), & x \geq E_{sw} \\ RE_0\, e^{-(x-E_{sw})/E_0}, & x < E_{sw} \end{cases} \qquad \text{Eq. (S1).}$$

Here, x represents the incident energy, R denotes the "ON" state reflectance, and E_{sw} denotes the switching energy. The ELU function offers a better depiction of the gradual switching process of

the NOMA cell compared to the ReLU function, which has a discontinuous first derivative at the threshold.

Based on the ELU fit, we extracted the switching energy E_{sw} as a function of V_c, which showed a linear dependence: $E_{sw} = 67V_c + 31$ (fJ) (Fig. S4(B)). The slope of the linear line is in good agreement with that obtained in pump-probe experiments (64 fJ/V, Fig. 2(D), inset). These results demonstrate the tunability of the implemented NOMA which can be useful in applications where incident light intensity varies, including all-optical neural networks and image processing. In these applications, the control voltage of the optical nonlinearity can be dynamically tuned to run computations under varying illumination conditions without having to substantially modify the optical system.

(d) Homogeneity of Optical ReLU: Using the same approach we outlined in the preceding section (SN2-c), we evaluated the response of NOMA at each individual pixel to quantify the pixel-to-pixel variations of the optical nonlinearity. For this purpose, instead of using a photodiode to integrate the total reflected energy, we used the CMOS camera and captured a wide-field image as shown in Fig. S5(A). The wide-field image contains 10,201 NOMA pixels. We segmented the wide-field image into grids (Fig. S5(A), red lines) that contain a single NOMA pixel and monitored the input-output relationship of each grid separately as shown in Fig. S5(B). For a given V_c and V_s, we captured the pixel-to-pixel variations using the switching energy as determined by the ELU fit (Eq. S1). The distribution of the switching energy is shown in the histogram of Figure 3(B) in main text. As shown in the color bar of Fig. S5(C), the switching energy across the 10,201 NOMA pixels was spatially uniform albeit with several defects which can be improved upon in future iterations of the fabrication process.

(e) Long-term Dynamic Stability: The long-term dynamic stability of the nonlinear layer is a more crucial factor, compared to static noise, for achieving high accuracy in ONNs, as dynamic noise—such as phase jitter in SLMs, power fluctuations in light sources, and drift in optical alignment—poses more significant challenges. To assess the long-term dynamic stability of the NOMA, we conducted an endurance test by periodically alternating the device between active and erase phases. During each active phase, an optical pulse with a duration of 50ms was illuminated onto four regions of NOMA. Each region had 10 NOMA pixels, representing a neuron. Every 20 cycles, a CCD camera captured the reflected image to monitor the CP reflected pulse energy, representing the neuron outputs. Over the 4000 switching cycles, the outputs of all neurons remained consistent (Fig. S6(A)). The inset histogram demonstrates that the dynamic deviation was below 0.33%. Furthermore, the ReLU-like optical nonlinear response of the four neurons was measured before and after the endurance test. As shown in Fig. S6(B), the nonlinear performance is almost identical, with a deviation of less than 1%, indicating a stable performance over time.

S3-Optical Neural Network Demonstration

(a) Setup of the Optical Neural Network: We designed a multilayer all-optical neural network (ML-AONN) that implements a two-layer fully connected neural network, specifically the mathematical operation $\boldsymbol{y} = \boldsymbol{W}^{(2)} f\left(\boldsymbol{W}^{(1)}\boldsymbol{x}\right)$. The optical layout of the AONN is illustrated in Figure S7. This setup consists of two matrix-vector multiplication (MVM) operations and one nonlinear activation function. The MVMs were implemented with two amplitude-only liquid crystal SLMs while the nonlinear activation was achieved using NOMA.

The first MVM computes the input for the hidden layer neurons as follows:

$$z = W^{(1)}x = \left(W_1^{(1)}, W_2^{(1)}\right)\begin{pmatrix} x_1 \\ x_2 \end{pmatrix} = W_1^{(1)}x_1 + W_2^{(1)}x_2 \qquad \text{Eq. (S2).}$$

where $W_1^{(1)}$ and $W_2^{(1)}$ represents the two columns of $W^{(1)}$. We encoded vector $W_1^{(1)}x_1$ and $W_2^{(1)}x_2$ separately onto two rectangle arrays displayed on the SLM1 (SDE1024, Cambridge Correlators, UK). Lenses L1 and L2, both with focal length of 20cm, were used to collimate the light reflected from the two rectangle array patterns. The two patterns were combined by a beam splitter (BS1) and projected onto NOMA with a 4X microscope objective. We ensured that the image on NOMA was an equal sum of intensities from each of the patterns $W_i^{(1)}x_i$ $(i = 1,2)$, thus optically implementing Eq. S2.

We implemented the nonlinear operation $a = f(z)$ by leveraging the ReLU-like response of NOMA, as depicted in Fig. 3(A) in the main text. Following the nonlinear activation, we employed optical fan-out to generate two copies of a using a multi-lens array (MLA). These copies were then imaged onto SLM2 (SXRD-211, SONY, Japan). SLM2 displayed two rectangle arrays with each array encoding the reflectance proportional to a row of $W^{(2)}$. Thus, the reflected light represents the element-wise multiplication between each row of $W^{(2)}$ and the output of the hidden layer, resulting in $s_1 = W_1^{(2)} \circ a$ and $s_2 = W_2^{(2)} \circ a$. The optical patterns of s_1 and s_2 were collimated and imaged onto a low noise CCD camera (PIXIS 400, Princeton Instrument, USA) using two lenses (L4 and L5), each with a focal length of 10cm. We captured the image and evaluated the values of s_1 and s_2 by averaging the intensity over the corresponding light spots. The final output scores, y_1 and y_2, were computed as the summation of s_1 and s_2, respectively. In our experiments, the light pulse has a duration of 62.5ms at a repetition rate of 10Hz. The CCD camera exposure time was 1.1s, integrating 10 pules per inference.

Currently the weighting in the first layer and the summation in the second layer are performed using a digital computer. With improved engineering, both operations could be implemented optically.

(1) Weighting operation in the first layer. This operation can be implemented optically by using two LEDs, where the intensity of each LED represents the one of the elements of the input vector $x = (x_1, x_2)$. Each LED illuminates a separate region of the SLM1, which displays two rectangular arrays. The reflectance of these arrays encodes the columns of the first layer weight matrix: $W_1^{(1)}$ and $W_2^{(1)}$. The reflected light from these arrays thus results in an intensity distribution proportional to the weighted outputs, $W_i^{(1)}x_i$ $(i = 1,2)$.
(2) Summation operation in the second layer. This operation can be implemented optically with a cylindrical lens that focuses the vertically aligned rectangular patterns of s_i into a line, whose intensity corresponds to the summation of s_i."

Although some operations in our ML-ONN are currently carried out electronically, our setup successfully demonstrates key optical operations, including optical summation (layer 1), optical weighting (layers 1 and 2), and optical fan-out (layer 2). Most importantly, with the NOMA serving as the nonlinear layer, there is no need to convert optical signals into the digital domain for nonlinear operations and then back into the optical domain. This capability enables true cascaded optical signal processing, paving the way for scalable deep optical neural networks.

(b) Linear Layer Calibration: Due to potential deviations from ideal behavior caused by nonuniform illumination and imaging aberrations, we first calibrated the two linear operations. For the first linear layer, we applied an element wise correction matrix C_1 to the true weight

matrix $\boldsymbol{W}^{(1)}$, resulting in a calibrated weight matrix $\boldsymbol{W}^{(1)'} = \boldsymbol{C_1} \circ \boldsymbol{W}^{(1)}$. We found the appropriate values of the correction matrix by replacing the NOMA with a CCD camera to directly image the output of the first linear layer. For the second linear operation, we employed a similar approach using calibration matrix $\boldsymbol{C_2}$ resulting in $\boldsymbol{W}^{(2)'} = \boldsymbol{C_2} \circ \boldsymbol{W}^{(2)}$. To test the precision of the linear operations, we conducted an MVM experiment. We set $\boldsymbol{W}^{(1)}$ as an all-ones matrix and generated 80 random $\begin{pmatrix} x_1 \\ x_2 \end{pmatrix}$ and $\boldsymbol{W}^{(2)}$. We used these values to calculate $\begin{pmatrix} y_1 \\ y_2 \end{pmatrix} = \boldsymbol{W}^{(2)}\boldsymbol{W}^{(1)} \begin{pmatrix} x_1 \\ x_2 \end{pmatrix}$ to form our ground truth. Experimentally, we ensured that NOMA was operating in a linear regime by setting V_c = 0V, V_s = 3.6V and ran MVMs with the calibrated weight matrices to get experimental outputs. The relation between experimental outputs and the ground truth for the linear operations is depicted in Fig. 4(B). The error rate of the two linear operations after calibration defined by a root mean squared error (RMSE) is 1.2%, which corresponds to a noise equivalent bit of 6.2bit for the linear operations ($NEB = -\log_2 RMSE$).

<u>(c) Nonlinear Layer Calibration:</u> Next, we calibrated the nonlinear response of NOMA. For these experiments, we operated NOMA at V_s = 3.6V and V_c = 5.5V. We set $\boldsymbol{W}^{(1)}$ and $\boldsymbol{W}^{(2)}$ as an all-ones matrix and set the input vector $\boldsymbol{x} = \begin{pmatrix} 1 \\ 1 \end{pmatrix} x$, where x was scanned from 0 to 1. Thus, the input of hidden neurons varied from 0 to 2. We measured the output of hidden neurons with the CCD camera. As shown in Fig. S8(A), the output of the four neurons behaves almost identically, indicating a uniform response of NOMA. To fit the nonlinear response, we use a modified ELU function as shown in Eq. (S3). Compared to the ELU function Eq. (S1), the modified ELU function adds a quadratic term in both the linear and the exponential portion of the ELU, which results in a better fitting result (Fig. S8(B)).

$$f(x) = \begin{cases} a_1(x - x_0) + a_2(x - x_0)^2 + b, \; x > x_0 \\ be^{\frac{/0}{1}(x-x_.)-p(x-x_.)^{\%}}, \; x < x_0 \end{cases} \qquad \text{Eq. (S3)}$$

<u>(d) ML-ONN Training and Weight Fine-Tuning:</u> The training of ML-ONN consists of two parts: (1) digital training and (2) weight fine tuning with the hardware feedback.

We first digitally trained a neural network with the same structure as our ML-ONN (two input neurons, four hidden neurons, and two output neurons). The nonlinear activation function was SEq. (3) with parameters determined by the curve fitting shown in Fig. S8(B). During the training, we constrained the weight matrix $\boldsymbol{W}^{(1)}$ and $\boldsymbol{W}^{(2)}$ as non-negative. We used cross-entropy loss and Adam optimizer to update the weights. Table S2 summarizes the hyperparameters used to train on the two datasets (XOR and circle). Post-training, we assessed the robustness of the weights at each epoch by evaluating inference accuracy across various computational precisions. We selected the weights demonstrating a high inference accuracy, particularly those performing well at a 4-bit precision, for implementation in the ML-ONN.

After completing digital training, we applied the trained weights for both layers obtained by the digital model and measured the experimental outputs of the second layer for the training data points, denoted as $\boldsymbol{s}_1^{exp}$ and $\boldsymbol{s}_2^{exp}$. We noted that the experimental values deviated from the theoretical values ($\boldsymbol{s}_1^{thy}$ and $\boldsymbol{s}_2^{thy}$) calculated by the digital model, but maintain a linear relationship, as shown in Fig. S9B. To address this mismatch, we perform a linear fitting between the experimental and theoretical values: $s_{1i}^{thy} = C_{1i}s_{1i}^{thy}$ and $s_{2i}^{thy} = C_{2i}s_{2i}^{thy}$. Then we modify the weight matrix of the second layer as $\boldsymbol{W}_{modified}^{(2)} = \boldsymbol{C} \circ \boldsymbol{W}^{(2)}$, where $\boldsymbol{C}$ is the fitted

coefficient matrix. After the weight fine-tuning the measured outputs showed good alignment between the experimental and theoretical values, as demonstrated in Fig. S9D.

Our fine-tuning approach is similar to a layer-by-layer fine-tuning process using experimentally collected data, which has been successfully implemented in previous optical neural networks training processes. This method effectively compensates for static non-uniformities in the optical setup and converges rapidly, eliminating the need for exhaustive parameter searches. Additionally, our digital model is finely calibrated to match the physical response of our NOMA, by ensuring that (i) without nonlinearity, the two-layer neural network performs matrix-vector multiplications with high precision, and (ii) The nonlinear behavior of the hidden neurons in our digital model closely mimic the actual behavior of the device.

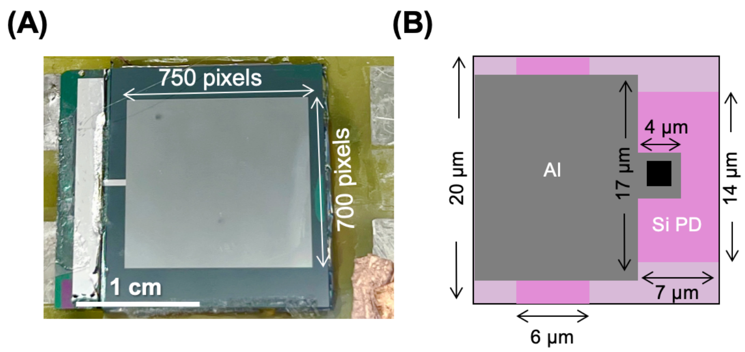

Fig. S1.

(A) Photograph of a completed NOMA with 750×700 pixels. **(B)** Top view of the NOMA design.

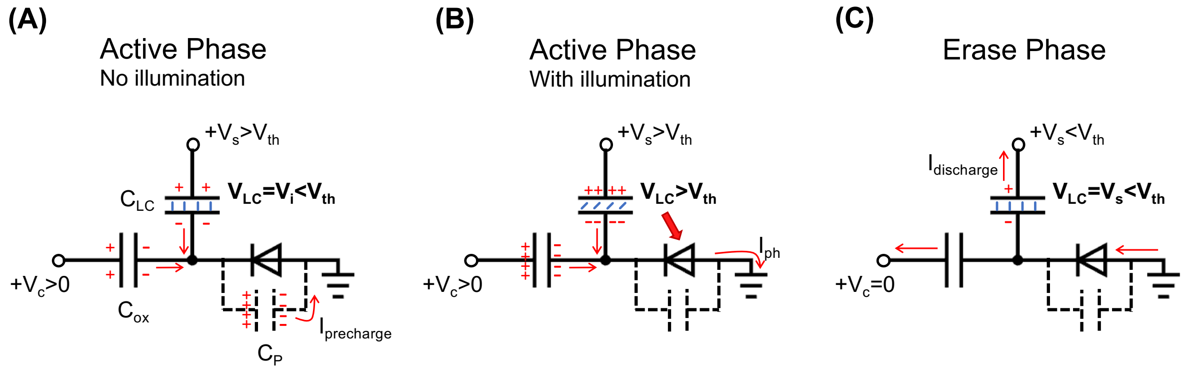

Fig. S2.

Circuit diagrams of NOMA in the active phase without illumination **(A)**, active phase with illumination **(B)**, and in the erase phase **(C)**, illustrating the change of the voltage on LC capacitor and the direction current flow.

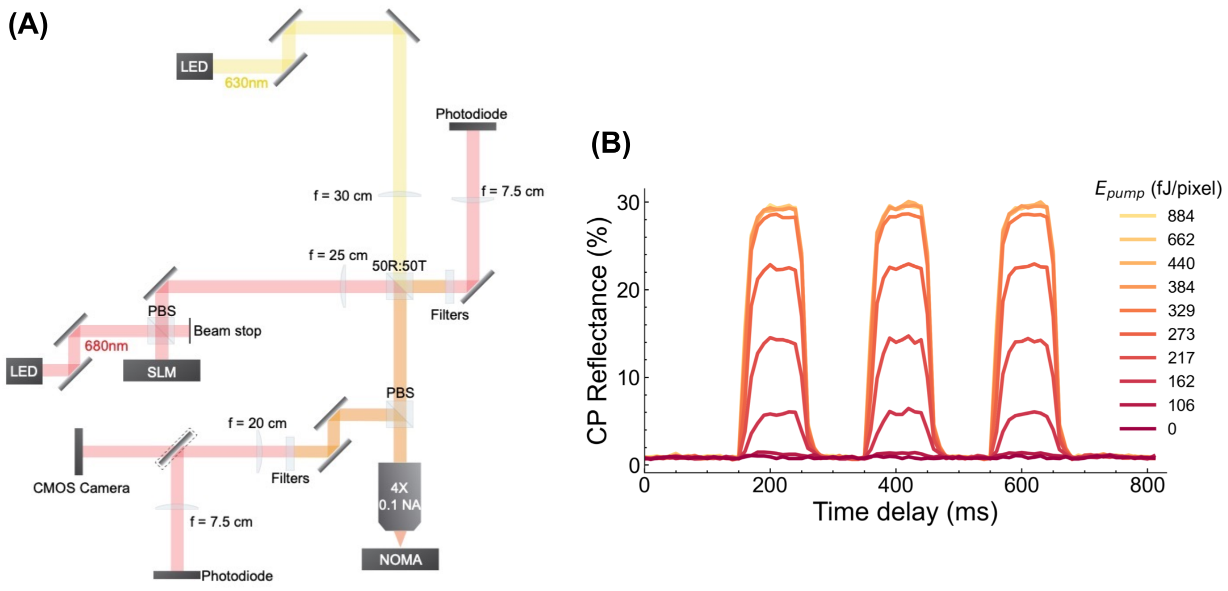

Fig. S3.

(A) Optical layout of the experiment built to characterize the nonlinear response of NOMA under two pulse and single pulse conditions**. (B)** Time traces of NOMA with three periods of active and erase phase at different pump energies.

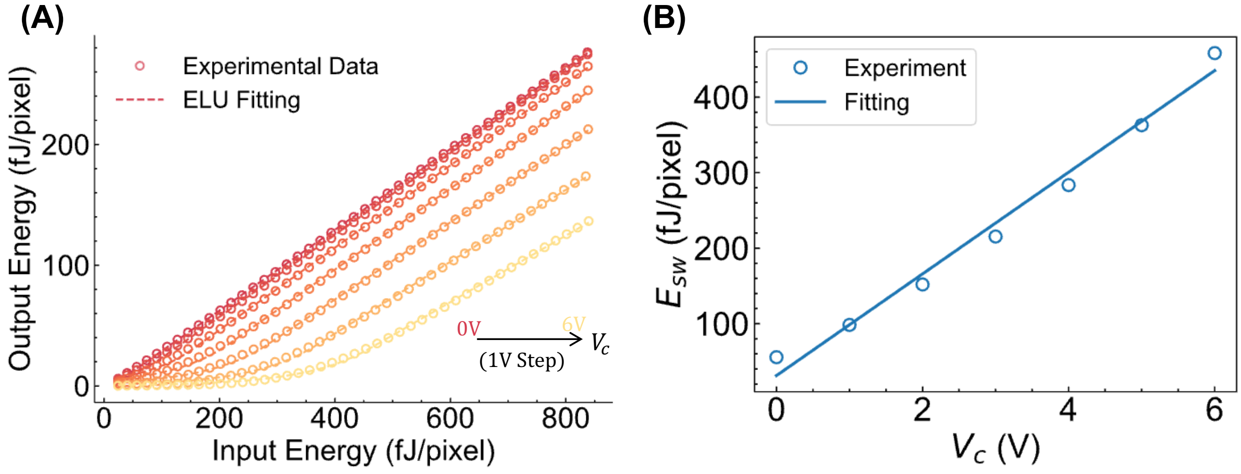

Fig. S4.

The ReLU-like optical nonlinear response of our NOMA device shows a dependence on V_c **(A)**, from which a linear relation between the ReLU threshold energy and V_c is extracted **(B)**.

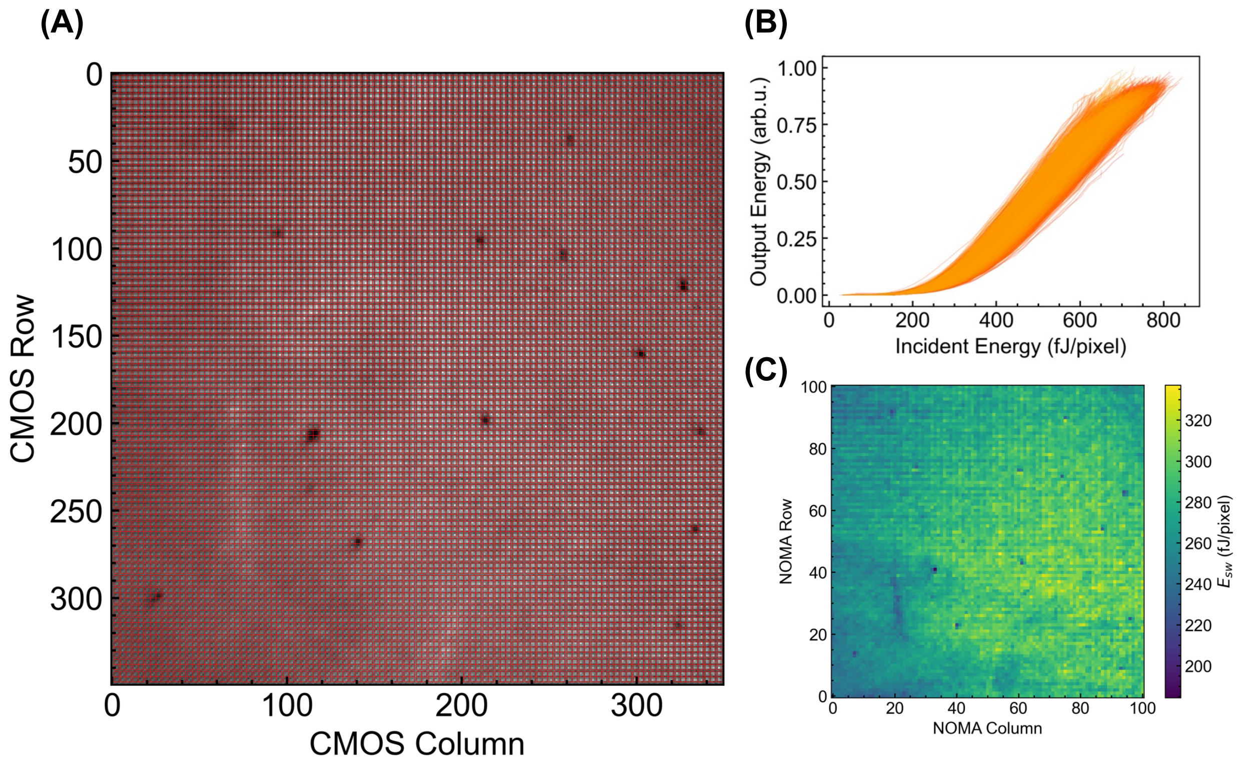

Fig. S5.

(A) The camera image of around 10200 NOMA pixels. We apply a grid method to identify each pixel for calculation of reflected intensity. **(B)** Optical nonlinear response of the 10200 NOMA pixels. **(C)** Threshold energy distribution of NOMA pixels.

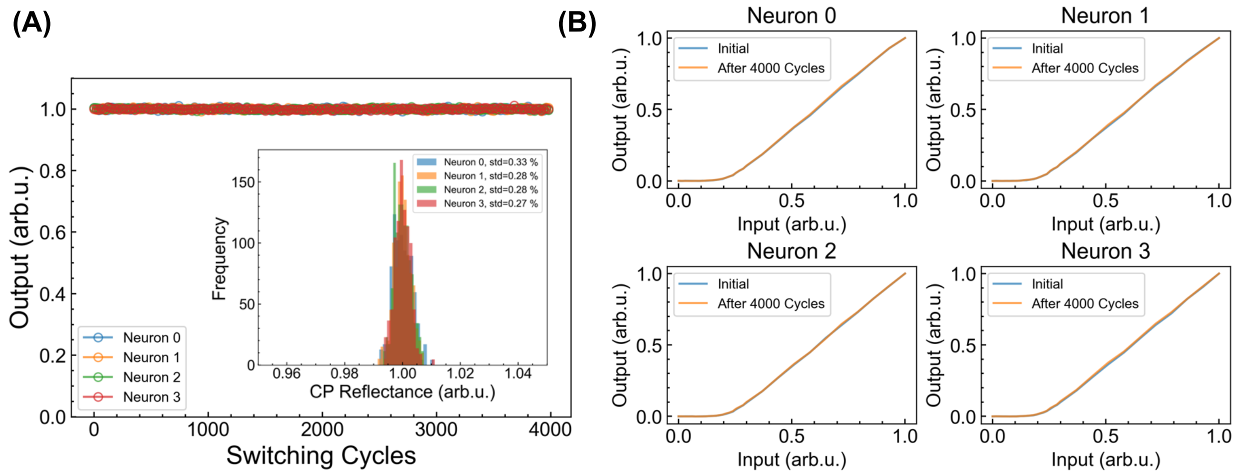

Fig. S6.

(A) Endurance test of four NOMA neurons. Insert shows the histogram the neuron outputs during the 4000 switching cycles. **(B)** ReLU-like optical nonlinear response of four NOMA neurons before and after the endurance test.

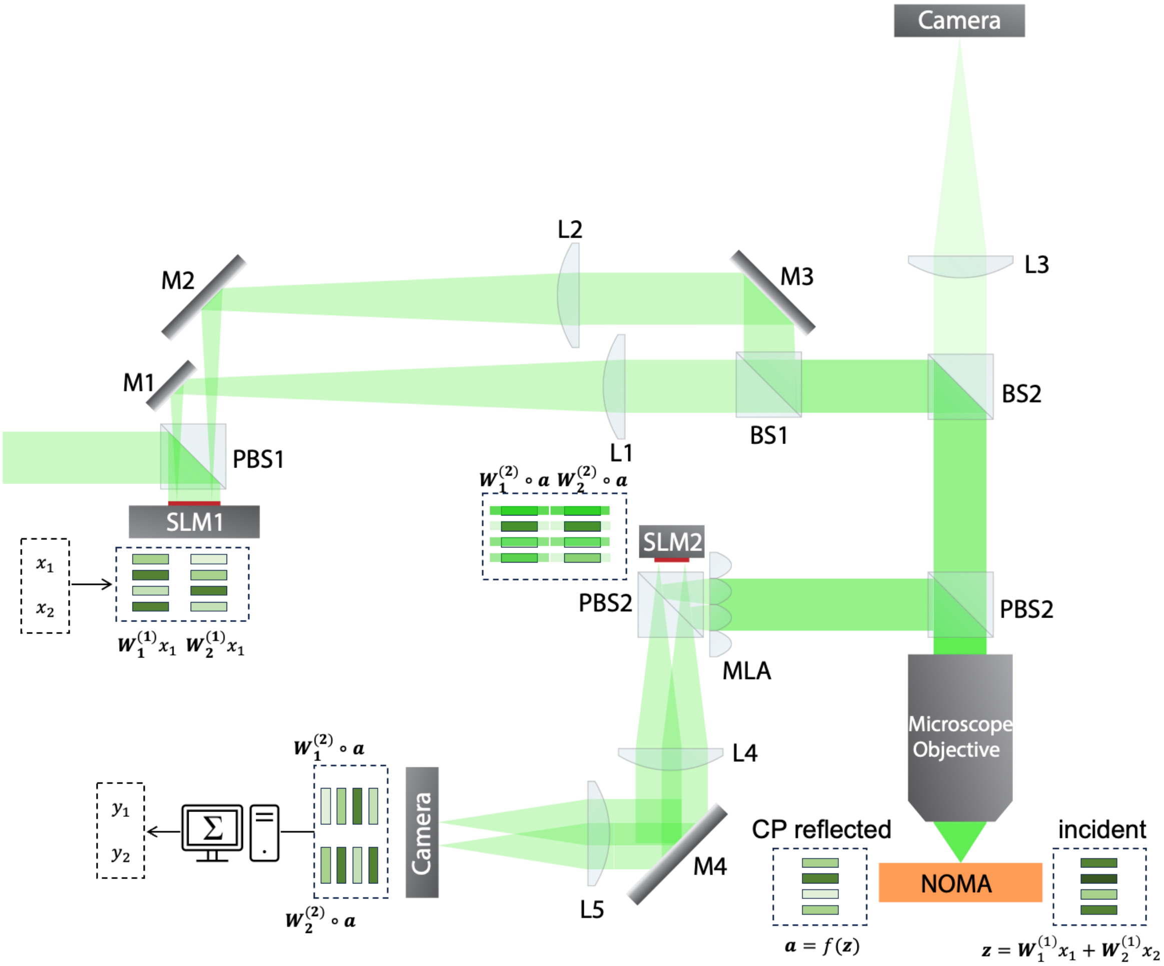

Fig. S7.

Optical layout of the implemented two-layer all optical neural network.

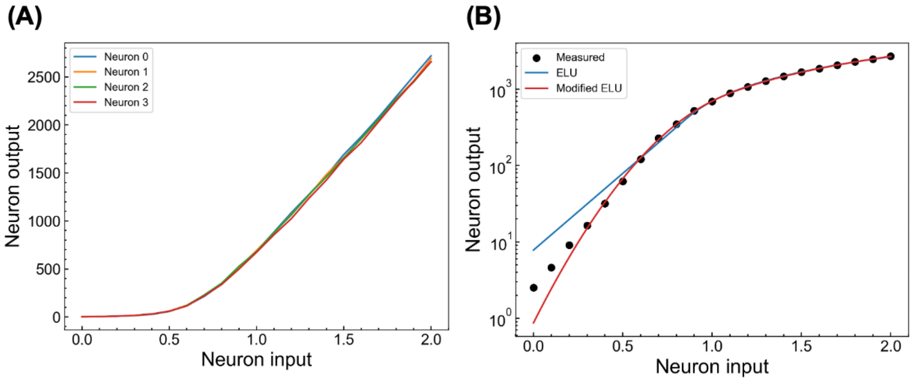

Fig. S8.

(A) Measured nonlinear response for each of the 4 neurons used in the ML-AONN. **(B)** The average neuron output is fitted with ELU and modified ELU function, showing in the logarithmic scale.

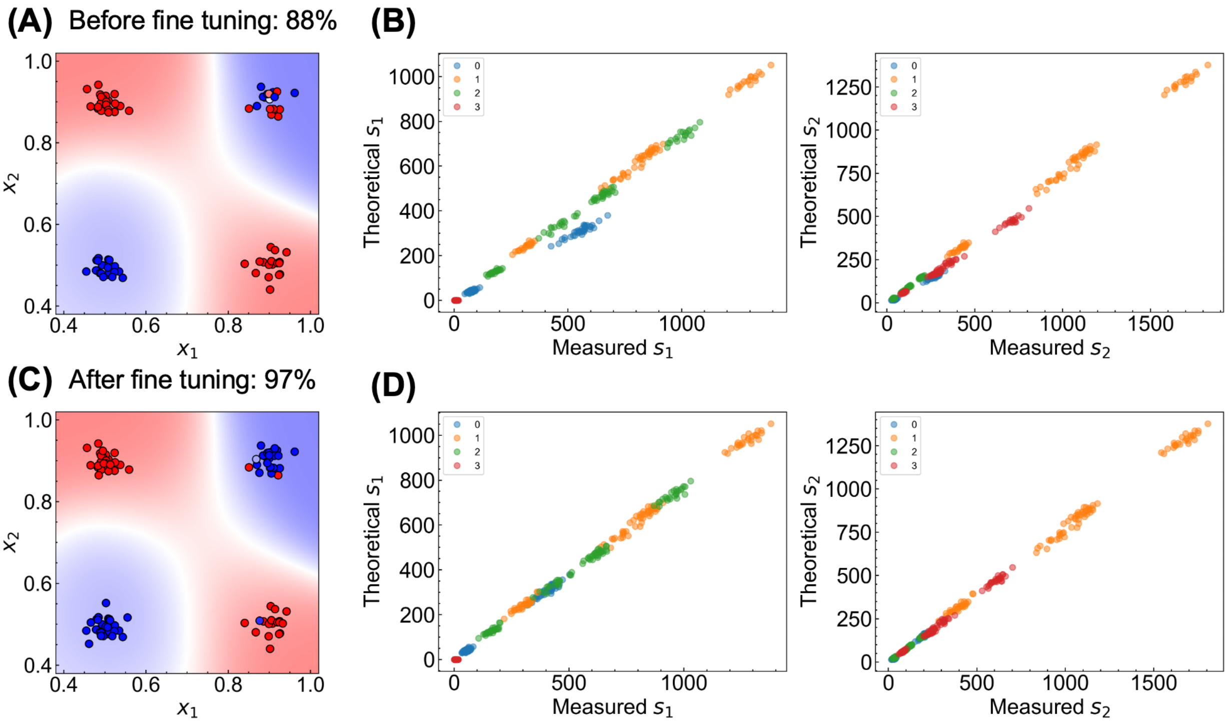

Fig. S9.

(A) Optical inference results before fine tuning. The measured values of s1 and s2 had discrepancies with their theoretical predictions **(B)**. After fine tuning the weights to compensate these discrepancies, the optical inference accuracy improved **(C)** and the measured values align better to the theoretical values **(D)**.

	Area (μm^2)	Dielectric constant	Thickness (µm)	Capacitance per pixel (fF)
C_{LC}	240	3.7-8.6 (LC)	3	2.6-6.1
C_{ox}	100	3.9 (SiO_2)	0.55*	6.4
C_p	140	11 (Si)	1.1**	12.4

* Effective oxide thickness
** Junction depth at zero reverse bias

Table S1.
Estimated capacitances of the NOMA.

	Training size	Test size	Optimizer	Batch size	Learning rate	Epochs
XOR	3200	800	Adam	400	0.05	200
Circle	3200	800	Adam	100	0.01	200

Table S2.
Hyper parameters used in digital training.
